# A comparison of leg length discrepancy between direct anterior and anterolateral approaches in total hip arthroplasty

**DOI:** 10.1186/s42836-020-00051-7

**Published:** 2020-11-02

**Authors:** Henry Dunn, Geoff Rohlfing, Robert Kollmorgen

**Affiliations:** UCSF Fresno, Fresno, CA USA

**Keywords:** Leg length discrepancy, Direct anterior approach, Anterolateral approach, Primary total hip arthroplasty, Intraoperative fluoroscopy, Overlay technique

## Abstract

**Background:**

Leg length discrepancy (LLD) after total hip arthroplasty (THA) is a known source of complications and a leading cause of litigation (J Bone Joint Surg Br 87:155–157, 2005). There are limited studies investigating surgical approach combined with the use of fluoroscopy intraoperatively and their potential effects on LLD after THA. The purpose of this study was to compare the direct anterior (DA) approach utilizing a fluoroscopic overlay technique and anterolateral (AL) approach and their potential effect on LLD.

**Methods:**

We retrospectively reviewed 121 patients who had undergone primary THA from September 1, 2016 to November 1, 2018 by either DA or AL approach by two separate surgeons. Leg length discrepancies were measured on pre-operative post-anesthesia care unit (PACU) and on post-operative low anterior/posterior (AP) pelvis plain radiographs by two investigators blinded to each other’s measurements. To confirm inter-observer and intra-observer reliability between LLD measurements amongst investigators, a Pearson correlation test was performed. The primary outcome measurement was leg length discrepancy (LLD).

**Results:**

We observed LLD > 1.0 cm and LLD > 1.5 cm in the DA and AL groups. The DA approach group showed a mean LLD of 4.5 mm against 7.76 mm in the AL group (*p* < 0.00001). There was a significantly higher rate of LLD in the AL group as compared to the DA group (LLD> 1 cm (28% *vs*. 8%, *p* = 0.0037) and LLD > 1.5 cm (7% *vs*. 0%, *p* = 0.0096). The LLD measurements showed strong correlation in terms of inter-observer (r = 0.95) and intra-observer reliability (r = 0.99) between the two investigators (*p* < 0.001).

**Conclusion:**

In our patient cohort, the DA approach with fluoroscopic overlay technique had less LLD in comparison with the AL approach, suggesting that intraoperative fluoroscopic use does have an impact on LLD.

## Introduction

Total hip arthroplasty (THA) is a successful and reliable procedure for pain relief as a treatment for advanced arthritis [1]. Patients reported forgotten hip joint score 70% at 1 year and 75% at 2 years [2]. Despite the success of this procedure, leg lengths that are not closely restored to equal result in leg length discrepancy (LDD). Consequently, leg length discrepancy is one of the top reasons for litigation following THA [3].

LLD after THA has been associated with overall dissatisfaction [3, 4] as well as gait disorders [5, 6], greater trochanteric pain [7], suspected aseptic loosening [8], and nerve palsy [9, 10]. Postoperatively, 32–42% of patients noticeg a difference in leg length [11, 12], and up to 45% required use of a shoe lift [11, 13]. Studies are conflicting as to what is acceptable LLD [3, 4, 14] and the degree to which LLD affects clinical outcomes [3, 14]. While leg length inequality of up to 20 mm can be asymptomatic, the amount of discrepancy causing symptoms varies [15], and discrepancy less than 10 mm has been believed not to produce symptoms and is well tolerated [4, 11].

Although LLD after THA cannot be eliminated, the problem can be minimized through preoperative templating, intraoperative examination and fluoroscopy, and, possibly, surgical approach [16]. Direct anterior-approach THA has become increasingly popular [17] and provides the opportunity to utilize intraoperative fluoroscopy to assess leg length and offset [18], while posterior and AL approaches offer spot films to asses intraoperative LLD.

Previous studies have shown that limb length measurements were similar between anterior and posterior approaches [19]. Evidence suggests that both the anterolateral and anterior approaches are able to safely, reliably and accurately produce optimal component positioning and leg length discrepancy [20–22].

Few previous studies examined the effect of surgical approaches on leg length discrepancy. Debi *et al* [23] compared acetabular positioning and leg length discrepancy between DA and AL approach, showing statistically significant difference in mean LLD between DA and AL. Bingham *et al* [24] didn’t find any difference in acetabular inclination, anteversion or LLD with use of intraoperative fluoroscopy in anterior hip arthroplasty. We hypothesize that, compared to the AL approach, the DA approach will produce less LLD when the intraoperative overlay technique is used.

## Materials and methods

We retrospectively reviewed patients who had undergone primary THA from September 1, 2016 to November 1, 2018 with the DA or AL. Institutional review board (IRB) approval was obtained (UCSF IRB #18–26,855) for the study and patient data were collected through the Research Electronic Data Capture, (REDCap). Inclusion criteria were patients who had undergone primary THA for treatment of primary osteoarthritis of the hip within a specified time range. Exclusion criteria included patients without sufficient radiographical data, those who had received contralateral THA, revision THA, and those without sufficient follow-up records. A power analysis showed that, a *p* < 0.05 was needed to have sufficient power; a minimum of 10 patients and a total of 100 patients were required. A total of 121 patients were included in the study, with 69 in the DA group and 52 in the AL group. Clinical outcomes, including post-operative infection, peri-prosthetic fracture, and Trendelenburg gait were recorded by reviewing patients’ charts. Demographics, such as age, BMI, sex, and ethnicity were also collected from patients’ charts.

**Fig. 1:**
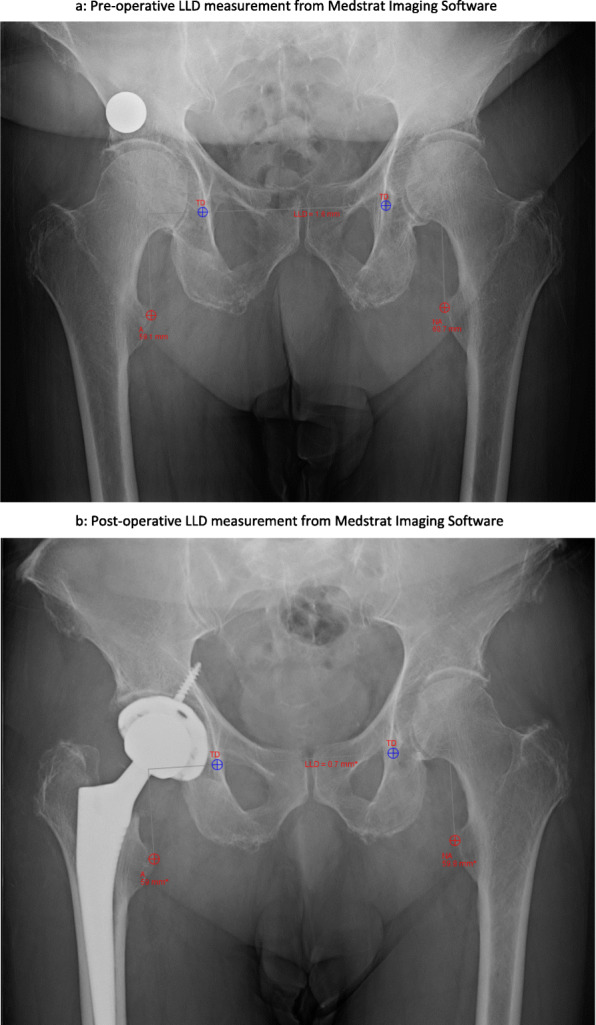
**a**, **b** Preoperative and postoperative AP pelvis XR with LLD measurement

Leg length difference was measured in office pre-operatively, in post anesthesia care unit (PACU), and in office 2 weeks after operation, all on low Anterior/Posterior (AP) pelvis plain radiographs. The trochanteric technique, as described by Dorr *et al* [25], was used to measure the LLD on the low AP pelvis XR (Fig. 1a and b). In brief, the tear drop line was marked bilaterally, creating a horizontal inter-tear drop line across the image. The distance between the inter-tear drop line and the most prominent portion of the lesser trochanter was measured and recorded [9].

**Fig. 2:**
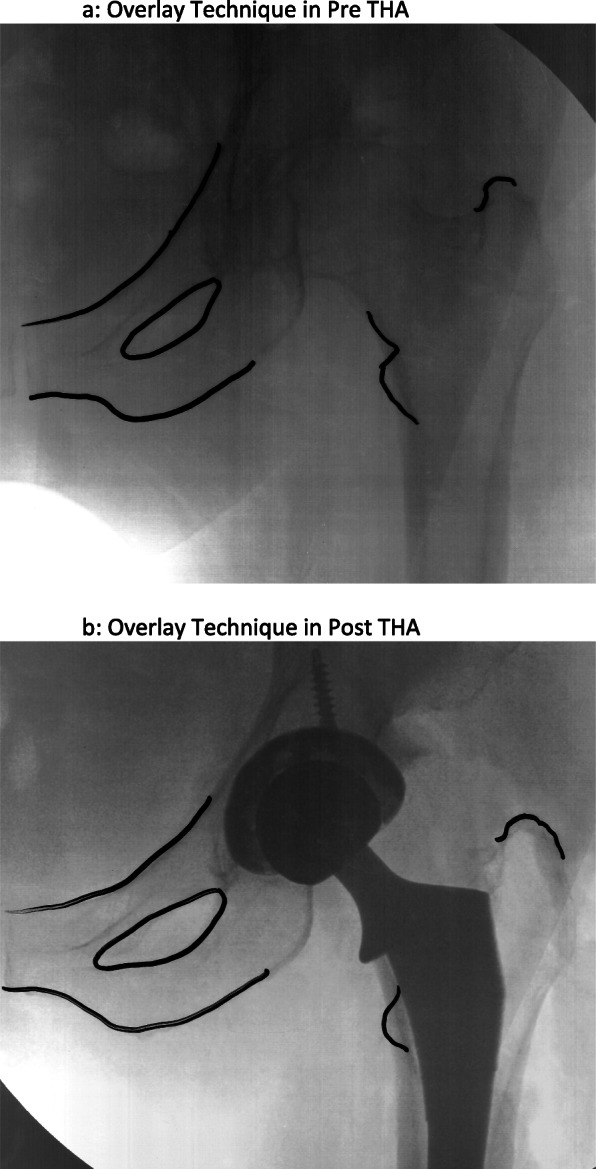
**a**, **b** Overlay Technique Pre and Post THA

Surgeries were performed via either DA or AL approach. The DA approach was performed with the technique described by Matta *et al* [20], with a specialized orthopaedic table used with the patient in supine position. Fluoroscopy was utilized for acetabular and femoral component placement. For this subset of patients, the overlay technique was employed for intraoperative LLD, as described by Olson *et al.* [18] In brief, fluoroscopy was used for acetabular component positioning and trial placement of the femoral component. A fluoroscopic image of the operative hip to include proximal femur and acetabulum was printed on a transparent film. A second image of the contralateral hip was also printed using the same image orientation and magnification. The two images could then be superimposed to assess the length and offset of the implanted prosthesis (Fig. 2).

The anterolateral approach was used in the lateral decubitus position on a standard operating table, as described by Hardinge [26]. With this approach, leg lengths were approximated by preoperative templating and neck length was verified in reference to the superior margin of the lesser trochanter. The femoral neck was osteotomized at the pre-planned level. Once the trial implants were in place, the hip was checked for stability before final implants were selected. Intraoperative XR or fluoroscopy was not used in the setting of the anterolateral approach.

Data were reviewed by two independent investigators who both utilized the technique described by Dorr *et al* [25] to measure LLD, with radiographic measurements recorded in REDCap. The operating surgeons (RK, DB) were excluded from the measurement process to ensure objectivity. To eliminate recall bias, each investigator performed radiographic measurements on each study participant twice, 2 weeks apart, with randomization of the participants on the second measurement, and with results recorded in REDCap. Statistical analysis was performed with SPSS software. Statistician from UCSF conducted the analysis. A two-tailed paired *t*-test was used to determine if the difference in means in leg length between the two groups was statistically significant (*p* < 0.05). Spearman rank correlations were calculated with R-value. Correlation in terms of R value was as follows: 1.0: linear, 0.7–1.0: strong, 0.3–0.7: moderate and 0–0.3: weak.

## Results

Mean age was 62.5 years (range: 32.6–87.2 years). Mean age was 60.6 in the DA group and 65 in AL group. The mean BMI was 30.14 (range: 17–41). Mean BMI was 29.97 in DA group and 30.38 in AL group. There were 59 males (48.76%) and 62 females (51.24%). There were 39 males and 30 females in the DA group and 20 males and 32 females in the AL group. The participants involved were 76% white (92), 22.3% hispanic [27], 1.65% African American [1]. Demographics are shown in Table 1. No differences were observed.

**Table 1:** Population characteristics

Approach	DA Group	AL Group	*p*-value
BMI	29.9	30.4	0.65
Age	60.6	65	0.37
Gender (M/F)	39/30	20/32	0.07

A total of 121 patients, 69 in the DA group and 52 in the AL group, were included in the study. The mean LLD was 4.52 mm in the DA group and 7.76 mm in the AL group (*p* < 0.05). There was a significant difference in LLD > 1.0 cm. The rate of LLD was 8% in the DA group and 28% in the AL group. In addition, there was a significant difference in LLD > 1.5 cm, which was 0% in the DA group and was 7% in the AL group. (Table 2).

**Table 2:** Leg length discrepancy

Approach	DA Group	AL Group	*p*-value
Mean LLD	4.5 mm	7.76 mm	< 0.0001
LLD > 1 cm	8.69%	28.80%	0.0037
LLD > 1.5 cm	0%	7.69%	0.0096

**Fig. 3:**
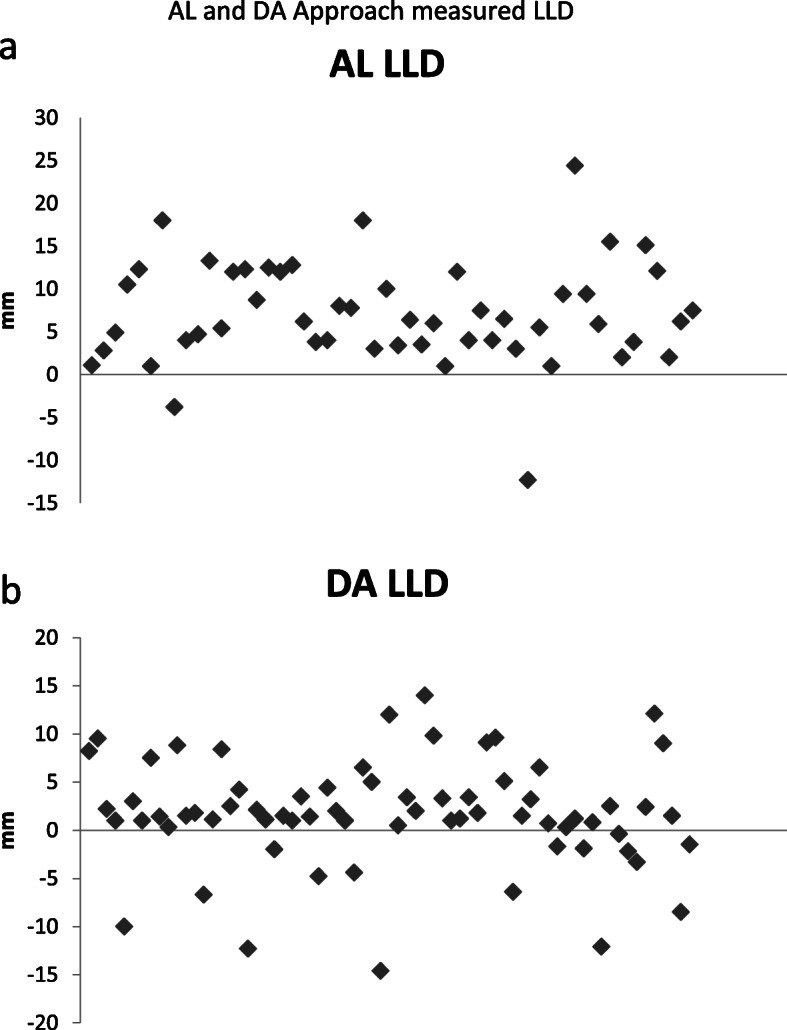
**a**, **b** Bland-Altman Plot: AL and DA Approach measured LLD in mm

The LLD measurements showed a strong correlation (r= > 0.70) in terms of inter-observer and intra-observer reliability. The Pearson correlation coefficient for the inter-observer analysis was 0.95, with *p*-value < 0.001. The Pearson correlation coefficient for the intra-observer analysis was 0.846, with *p* < 0.001 for the first observer, and the Pearson correlation coefficient was 0.99, with *p* < 0.001 for the second observer. Figures 3a and b show the individual LLD measurements (in mm) of the DA and AL approaches in Bland-Altman plots.

## Discussion

In our patient cohort, the DA approach had less average LLD in comparison with the AL approach, suggesting that surgical approach with use of fluoroscopy does have an impact on LLD. In terms of LLD > 1.0 cm, the rate of LLD was 8% in the DA group, and 28% in the AL group. In addition, no patient in the DA group had LLD > 1.5 cm and and 7% of the patients in the AL group had LLD > 1.5 cm.

THA uses three main approaches: anterior, anterolateral, and posterior. The anterior approach is routinely used in the patient in the supine position and allows for intraoperative fluoroscopy. Whereas the anterolateral and posterior approaches, which are performed with patients assuming the lateral decubitus position, generally do not use fluoroscopy intraoperatively. An explanation for the better accuracy of the DA group could be attributed to the intraoperative imaging that allows for immediate feedback to the surgeon. The AL approach is performed in the lateral decubitus position, which, if fluoroscopy was to be used, complicates the intraoperative acquisition of optimal fluoroscopic imaging. Patients lying lateral on a standard operating table for an AL approach may be rotated forward, and, if not secured properly, the surgeon might be misled on pelvic rotation and potential LLD [27]. The LLD can be estimated during AL approach with soft tissue tension or shuck tests but this may be affected by the neuromuscular blockade caused by anaesthesia used [28].

In addition, clinical palpation of bony landmarks in the lateral position may be misleading when the operated limb is in adduction, with a variation of 10°, resulting in up to 17 mm of measured LLD [28].

Debi *et al* [23] compared 172 patients undergoing THA by a single surgeon using the AL or DA approach with intraoperative fluoroscopy. They found that LLD was improved in patients undergoing THA through the DA approach with intraoperative fluoroscopy, compared to the AL approach. None of the DA patients had LLD > 6 mm, whereas 7.4% of the AL group had LLD > 6 mm, and 2.1% of the AL group had LLD > 10 mm. They concluded that the optimal control of LLD with intraoperative fluoroscopy with the DA approach was due to easier acquisition of fluoroscopic images with the patient in the supine position. Placing the patient in the supine position allows for the combination of both radiographical and clinical checking of the leg length, potentially leading to more accurate LLD. The results of their study were comparable to what we found in our patient population.

Beamer *et al* [27] reported 52 patients who underwent THA with freehand implant placement and 57 patients with use of intraoperative fluoroscopy. They found no significant difference in LLD in the groups with postoperative LLD < 5 mm, between 5 and 10 mm, 10–15 mm, or > 15 mm increments. Bingham *et al* [24] retrospectively reviewed 265 patients who underwent DA THA with or without the use of intraoperative fluoroscopy. They found no difference in LLD between the two groups, with mean LLD being 1.1 mm and 0.8 mm respectively. They concluded that equivalent radiographic outcomes are achievable without use of intraoperative imaging. They suggested that the accuracy resulted from the combination of high surgical volume, precise preoperative templating, and intraoperative clinical assessment. The LLD results in these studies were not affected by the use of fluoroscopy, suggesting that the DA approach for THA, independent of use of fluoroscopy, is extremely accurate. This accuracy is attributable to the reproducible intraoperative clinical assessment of LLD using the contralateral leg and external landmarks with the supine position using the DA approach. In the supine position, there is less concern for misleading LLD results due to operative limb adduction variation or pelvic rotation, as compared to the lateral decubitus position.

The study has potential limitations. Firstly, although the DA group had statistically significant decrease in radiographic leg length discrepancy (LLD), both methods achieved satisfactory clinical outcomes. The fact that only one surgeon used intraoperative fluoroscopy may be a limitation to our current study. Obtaining an intraoperative fluoroscopic image in the lateral position is more challenging and should be considered a limitation as well. Due to the retrospective nature of the study, we did not clinically measure the LLD or report patient outcome scores.

## Conclusion

Our study demonstrated that LLD was more accurate in patients undergoing THA with the DA approach with the use of intraoperative fluoroscopy in comparison to the AL approach.

## Data Availability

The datasets generated and/or analysed during the current study are not publicly available because the data set contained protected health information but are available from the corresponding author on reasonable request.

## Abbreviations

LLD: Leg Length Discrepancy
THA: Total Hip Arthroplasty
DA: Direct Anterior
AL: Anterolateral
PACU: Post Operative Anesthesia Care Unit
AP: Anterior/Posterior
IRB: Institutional Review Board
(REDCap): Research Electronic Data Capture
